# A tissue level atlas of the healthy human virome

**DOI:** 10.1186/s12915-020-00785-5

**Published:** 2020-06-04

**Authors:** Ryuichi Kumata, Jumpei Ito, Kenta Takahashi, Tadaki Suzuki, Kei Sato

**Affiliations:** 1 Division of Systems Virology, Department of Infectious Disease Control, International Research Center for Infectious Diseases, Institute of Medical Science, The University of Tokyo, 4-6-1 Shirokanedai, Minato-ku, Tokyo, 1088639 Japan; 2 Department of Pathology, National Institute of Infectious Diseases, Tokyo, 1628640 Japan; 3 CREST, Japan Science and Technology Agency, Saitama, 3220012 Japan

**Keywords:** Human virome, Transcriptome, GTEx, Human gene expression, Microbiome

## Abstract

**Background:**

Human-resident microbes can influence both health and disease. Investigating the microbiome using next-generation sequencing technology has revealed examples of mutualism and conflict between microbes and humans. Comparing to bacteria, the viral component of the microbiome (i.e., the “virome”) is understudied. Somatic tissues of healthy individuals are usually inaccessible for the virome sampling; therefore, there is limited understanding of the presence and distribution of viruses in tissues in healthy individuals and how virus infection associates with human gene expression and perturbs immunological homeostasis.

**Results:**

To characterize the human virome in a tissue-specific manner, here we performed meta-transcriptomic analysis using the RNA-sequencing dataset from the Genotype-Tissue Expression (GTEx) Project. We analyzed the 8991 RNA-sequencing data obtained from 51 somatic tissues from 547 individuals and successfully detected 39 viral species in at least one tissue. We then investigated associations between virus infection and human gene expression and human disease onset. We detected some expected relationships; for instance, hepatitis C virus infection in the liver was strongly associated with interferon-stimulated gene upregulation and pathological findings of chronic hepatitis. The presence of herpes simplex virus type 1 in one subject’s brain strongly associated with immune gene expression. While torque teno virus was detected in a broad range of human tissues, it was not associated with interferon responses. Being notable in light of its association with lymphoproliferative disorders, Epstein-Barr virus infection in the spleen and blood was associated with an increase in plasma cells in healthy subjects. Human herpesvirus 7 was often detected in the stomach; intriguingly, it associated with the proportion of human leukocytes in the stomach as well as digestive gene expression. Moreover, virus infections in the local tissues associated with systemic immune responses in circulating blood.

**Conclusions:**

To our knowledge, this study is the first comprehensive investigation of the human virome in a variety of tissues in healthy individuals through meta-transcriptomic analysis. Further investigation of the associations described here, and application of this analytical pipeline to additional datasets, will be useful to reveal the impact of viral infections on human health.

## Introduction

Advances in next-generation sequencing (NGS) methods in recent decades have made comprehensive surveys of a variety of microorganisms possible. Metagenomic analyses have explored microorganisms, including bacteria, phages, and viruses, in a variety of places, such as the oceans [1–4] and soils [5–7] and on Earth [8]. Perhaps, the most deeply surveyed microbiome is that of humans. The Human Microbiome Project aims to characterize bacteria, viruses, and other microorganisms in the human body [9, 10]. As they often live outside human cells, the human bacterial microbiome has been well described in multiple organs and samples via non-destructive sampling: the bacterial microbiome of the skin [11, 12], oral cavity [13], and gastrointestinal tract (including feces) [14] are well described. A major theme emerging from these studies is that, while certain microbial species are associated with pathology, many components of the microbiome likely play a symbiotic role in maintaining human health (reviewed in [15–18]).

Many viruses are clearly human pathogens: human immunodeficiency virus (HIV) and influenza virus are the causative agents of human diseases, and Epstein-Barr virus (EBV; also known as human herpesvirus 4 [HHV-4]) [19] and hepatitis C virus (HCV) [20] can drive oncogenesis. On the other hand, similar to the cases of the human bacterial microbiome, some viruses can chronically infect a broad range of human tissues without overt pathology. Previous studies have suggested that these viruses nonetheless have detrimental effects. For example, human respiratory syncytial virus and human rhinoviruses may play an important role in the inceptions of childhood asthma and atopic asthma, respectively [21, 22]. On the other hand, there are a few examples of viruses that exhibit protective effects. For example, GB virus C (GBV-C, also known as hepatitis G virus [HGV]) infection can be protective against HIV infection and improve survival [23]. In experiments using animal models, latent infection with murine gammaherpesvirus, which is genetically related to EBV in humans, confers symbiotic protection from bacterial infection [24]. Thus, virus infections are associated with multiple aspects of human health, and revealing the human “virome” would be beneficial for understanding the hidden mutualism and/or conflict between humans and viruses [25]. In the largest study of the human virome to date, Simon et al. performed meta-transcriptome analysis using more than 17,000 human RNA-sequencing (RNA-Seq) datasets to reveal the human virome [26]. However, the dataset used in this previous study is related to human diseases. Moustafa et al. used DNA-sequencing data obtained after whole-genome sequencing of more than 8000 “healthy” subjects using blood samples. The authors detected 19 human viruses in the blood; however notably, their approach was “blind” to RNA viruses [27]. Previous studies of the human virome have used specimens that are relatively easy to access in healthy individuals (e.g., blood or skin). In other words, it is technically difficult to obtain the somatic tissues from inside the human body (e.g., brain and internal organs) of healthy individuals for virome investigation. Moreover, it remains unclear (1) what kind of viruses infect in various tissues in healthy individuals and (2) how these virus infections influence human gene expression and perturb the homeostasis of these tissues.

To characterize the virome in the human body, we performed meta-transcriptomic analysis using RNA-Seq dataset provided by the Genotype-Tissue Expression (GTEx) Project [28]. We detected 39 viruses in a variety of human tissues, revealing both expected and unexpected associations between viral infections and human gene expression and human disease.

## Results

### The human virome across tissues

**Fig. 1:**
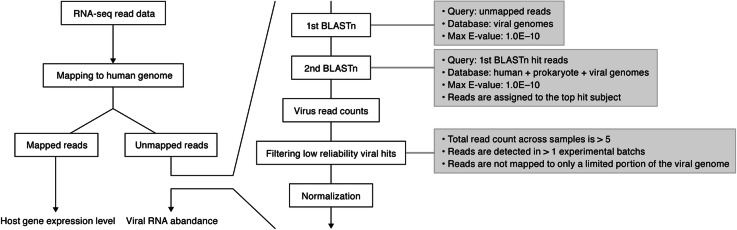
Workflow of this study. Flow diagrams of the meta-transcriptomic analysis pipeline constructed in this study. A detailed description is provided in the “Methods” section

**Fig. 2:**
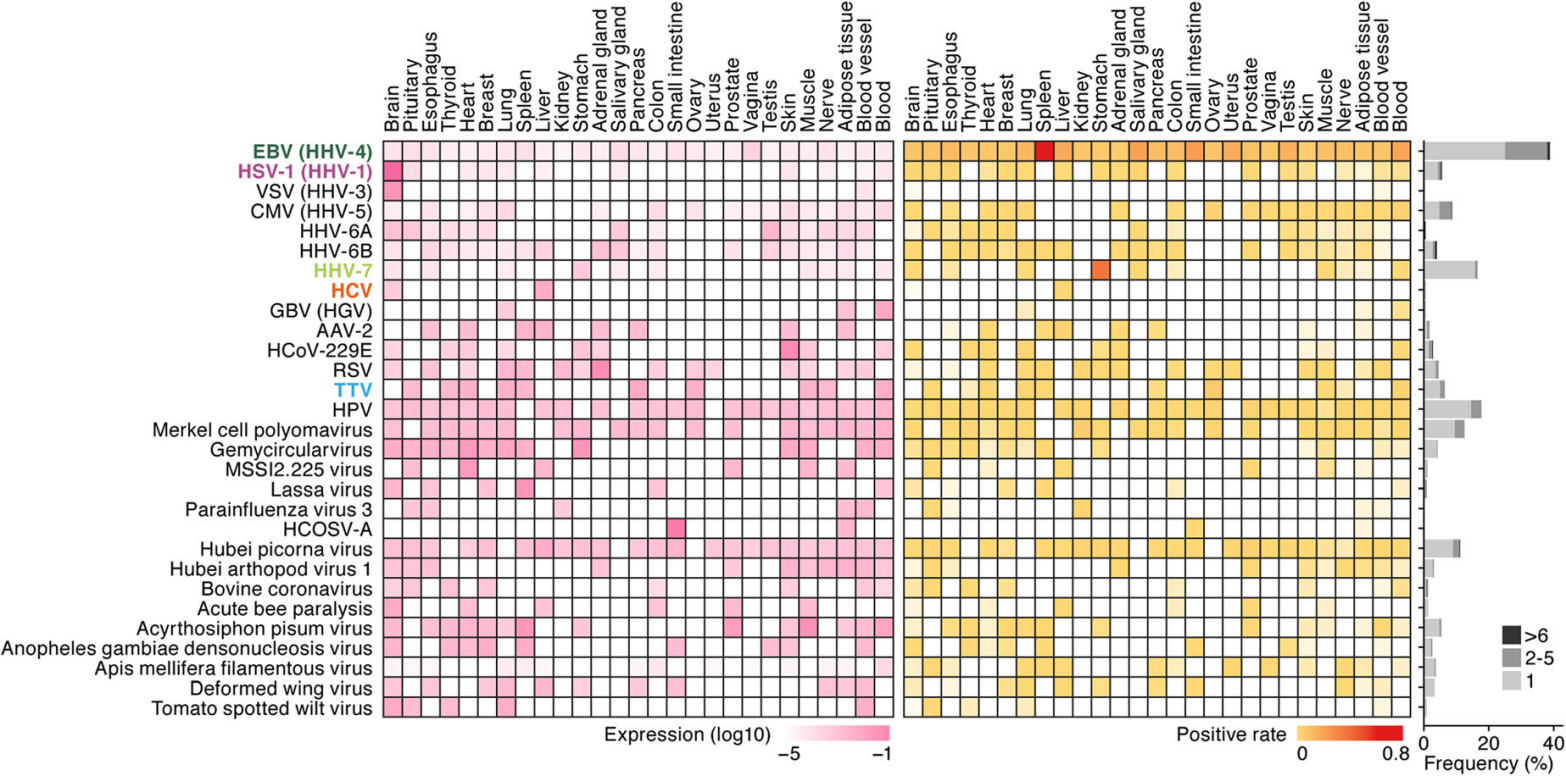
Landscape of the healthy human virome. The viruses detected in the GTEx dataset are summarized. The results of various subtypes of TTVs, HPVs, and Hubei picornaviruses detected were unified. The viruses for further investigations in Figs. 3, 4, 5, 6, and 7 (EBV, HSV-1, HHV-7, HCV, and TTV) are indicated in bold and colored. The alternative name of each virus is indicated in parentheses. (Left) The level of the transcript of each virus in each human tissue. The averages in the positive samples are shown as a heatmap. (Middle) The positive rate for each virus in each human tissue. (Right) The frequency of the individuals positive for respective viruses is summarized in bar graphs. The numbers of tissues positive for the respective viruses (1, 2–5, or more than 6) are also shown. AAV, adeno-associated virus; HCoV, human coronavirus; RSV, respiratory syncytial virus

To characterize the virome across the human body, we analyzed the 8991 RNA-Seq data obtained from 51 somatic tissues of 547 individuals provided by the GTEx Project [28] (Additional file 1: Table S1). Using a new analytical pipeline (Fig. 1), we screened for viral RNAs derived from 5139 viruses and measured their abundances in the RNA-Seq dataset (see the “Methods” section). We detected 39 viral species (Fig. 2 and Additional file 2: Table S2 and Additional file 3: Table S3). We detected human herpesviruses, including EBV, herpes simplex virus type 1 (HSV-1; also known as human herpes virus 1 [HHV-1]), varicella-zoster virus (VZV; also known as human herpes virus 3 [HHV-3]), cytomegalovirus (CMV; also known as human herpes virus 5 [HHV-5]), human herpesvirus 6A (HHV-6A) and 6B (HHV-6B), human herpesvirus 7 (HHV-7), and other human viruses such as HCV and torque teno virus (TTV) (Fig. 2). Consistent with a previous study [27], we also detected non-human viruses, including insect viruses (e.g., deformed wing virus) and a plant virus (e.g., tomato spotted wilt virus). These non-human viruses may reflect “contamination” at the tissue level (e.g., plant matter in the gastrointestinal tract), during library preparation/sequencing or potentially, unexpected tropism of these or related viruses.

Some human viruses (e.g., EBV and human papilloma virus [HPV]) were detected in a broad range of tissues in positive individuals, while the other viruses, including HCV and HHV-7, were detected in a tissue-specific manner (Fig. 2, right; for additional detail, see the subsequent sections). While we detected 16 RNA viruses, most detected human viruses were DNA viruses, including herpesviruses. Overrepresentation of DNA viruses in the human virome has previously been considered to result from sampling bias, as a previous study of the human virome has used DNA-sequencing [30]. However, our results suggest that mainly DNA viruses shape the healthy human virome even at the transcriptome level. We cannot fully exclude the possibility that the detection sensitivity of RNA viruses is lower than that of DNA viruses, perhaps due to the lower sequence conservation of RNA viruses compared to DNA viruses [31], or the acute nature of many RNA virus infections.

Another possibility for the abundant detection of human herpesviruses (e.g., EBV, HHV-6, and HHV-7) is that reads were dominantly mapped to telomeric/repetitive sequences of these viruses [19, 32]. Although the telomeric/repetitive regions were not covered by the masked regions completely, only a limited portion of viral-assigned reads were mapped to these regions (Additional file 4: Figure S1 and Additional file 5: Table S4). Therefore, we concluded that the presence of viral telomeric/repetitive regions does not affect our computational pipeline to measure viral abundance and that the human herpesviruses detected are authentic.

### Association of HCV infection with robust interferon responses in the liver and the onset of hepatitis

**Fig. 3:**
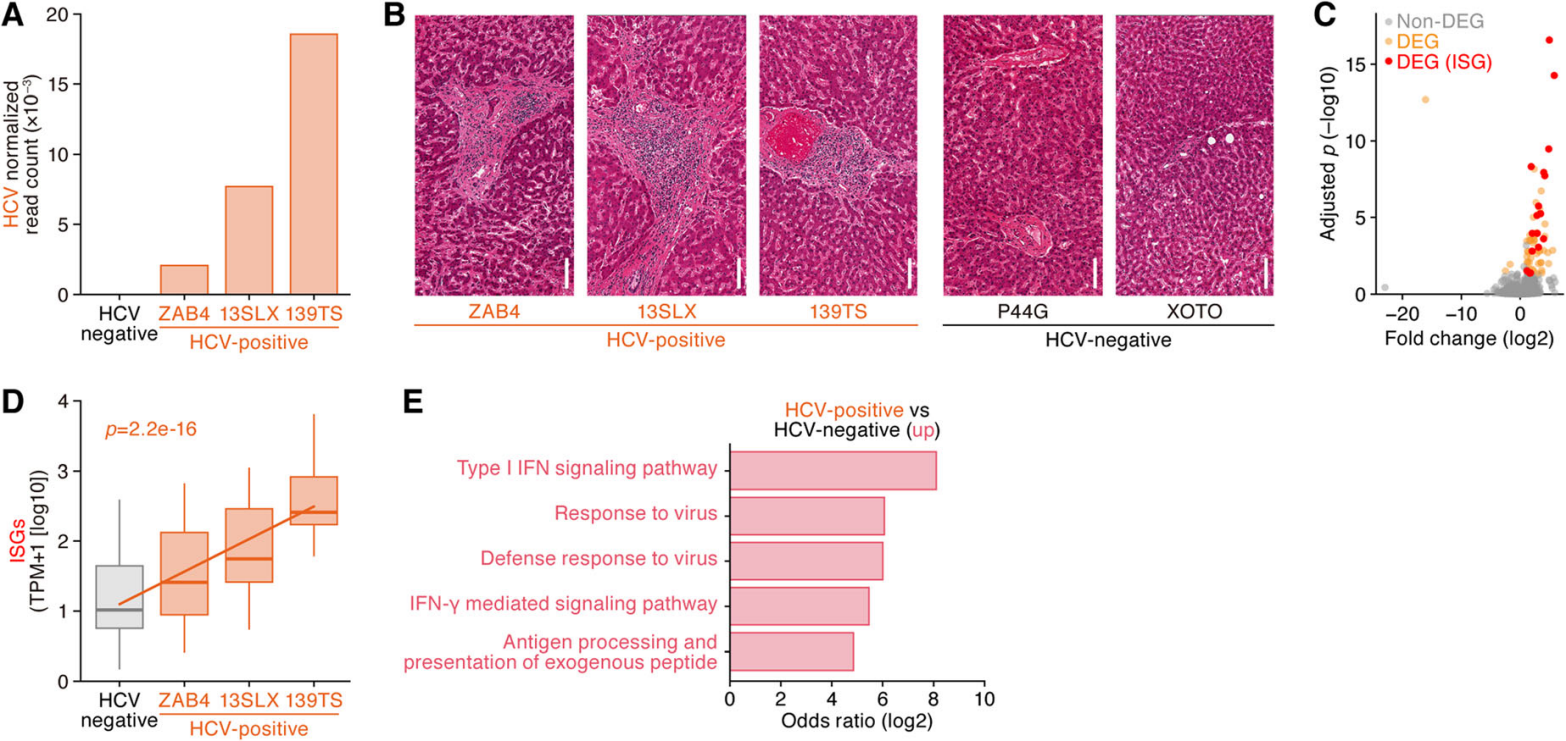
HCV in the livers with chronic hepatitis. **a** Detection of three individuals positive for HCV transcripts in the liver. The normalized read counts of HCV in the respective samples (sample IDs: ZAB4, 13SLX, and 139TS) are shown. **b** Histological observations in the liver of the three HCV-positive individuals. The photos of hematoxylin and eosin staining are provided from the GTEx dataset [29]. Two HCV-negative livers (sample IDs: P44G and XOTO) are shown as the negative controls. **c** Differential expression of genes between HCV-positive and HCV-negative livers. The *X*-axis indicates the fold change score, and the *Y*-axis indicates the statistical score. The positive and negative fold change scores indicate the up- and downregulations in the HCV-positive livers, respectively. The DEGs are indicated in pink dots, and ISGs are indicated in red with each gene symbol. **d** Expression levels of the upregulated ISGs in the three HCV-positive livers. Statistical significance was evaluated by linear regression test. **e** GO enrichment analysis of the upregulated genes in HCV-positive livers. The statistical summary of distribution is shown as a box plot. The terms with statistical significance (FDR < 0.05) are shown

Importantly, our approach allows us to quantify both viral RNA and host gene expression in the same sample (Fig. 1). We thus analyzed these two quantities to explore the effect of each virus on human gene expression patterns, comparing virus-positive samples to virus-negative samples in the same tissue. We first focused on HCV, which was specifically detected in liver (Fig. 2). HCV is classified into the family *Flaviviridae*, genus *Hepacivirus* and possesses a positive-sense single-stranded RNA (~ 10 kb) genome [20]. HCV is a causative agent of human hepatitis, and chronic infection with HCV can lead to severe illnesses including liver cirrhosis and hepatocellular carcinoma [20]. HCV was detected almost only in the liver (Fig. 2) and was found in three (GTEx sample IDs: ZAB4, 13SLX, and 139TS; see also Additional file 6: Table S5) out of 136 subjects with liver sampled (Fig. 3a). In histopathological examination, all three HCV-positive hepatic tissues showed portal tract expansion with fibrosis, bile duct reactive change, and lymphocyte infiltration and aggregation (Fig. 3b, left). Interface hepatitis and bridging fibrosis were also observed in two (13SLX and 139TS) and one (13SLX) case(s), respectively (Fig. 3b, left). These histological findings are compatible with hepatitis. On the other hand, two HCV-negative liver samples did not show these morphologic features suggesting hepatitis (Fig. 3b, right). Although hepatic disease did not cause their death, our findings suggest that HCV infection contributed to undiagnosed hepatitis in these three individuals.

HCV infection is sensed by cellular pathogen recognition receptors (PRRs) and can cause interferon (IFN) production. IFN-mediated signaling leads to expression of IFN-stimulated genes (ISGs), many of which exhibit antiviral effects (reviewed in [33, 34]). To address whether HCV infection is associated with IFN responses, including ISG upregulation in this dataset, we assessed the expression levels of ISGs in the liver. As shown in Fig. 3c, 18 out of the 62 genes significantly upregulated in HCV-positive compared to HCV-negative livers were ISGs. Moreover, the abundance of HCV RNA (Fig. 3a) clearly correlated with the expression levels of these 18 ISGs (Fig. 3d; *p* = 2.2e−16 by linear regression test). Moreover, gene ontology (GO) enrichment analysis of the differentially expressed genes (DEGs) between HCV-positive and HCV-negative livers indicated that the genes associated with “type I interferon signaling pathway,” “defense response to virus,” and “viral process” were highly upregulated in the HCV-positive livers (Fig. 3e). Altogether, these findings are consistent with previous findings [33, 34] and verify the biological significance of the results by our analytic pipeline.

Based on the following two hallmarks, histological observations suggesting hepatitis (Fig. 3b) and upregulation of ISGs (Fig. 3c and d), the detection of HCV reads is a good chance to validate the specificity and sensitivity of our analytical pipeline. To validate the performance of our computational pipeline, we compared ours with the other three computational pipelines, Kraken [35], CLARK [36], and Kaiju [37], which have been previously reported as computational pipelines to identify viral sequences from NGS data. As shown in Additional file 7: Figure S2, these three pipelines detected HCV reads from the three HCV-positive samples (sample IDs: ZAB4, 13SLX and 139TS) identified by our pipeline. However, CLARK and Kraken detected tremendous amounts of HCV reads from all liver samples (Additional file 7: Figure S2). Taken together with the fact that the prevalence of HCV infection worldwide is less than 5% [38], these results suggest that the results obtained by CLARK and Kraken contain many false-positive hits, and these pipelines are relatively less specific for at least HCV in the dataset used in this study. On the other hand, the result obtained by Kaiju was similar to that by our pipeline (Additional file 7: Figure S2). Collectively, these results strongly support the validity of our analytical pipeline.

### EBV infection in human plasma cells

We next focused on EBV, the most broadly detected virus in multiple individuals (Fig. 2, right). EBV is classified into the family *Herpesviridae*, genus *Lymphocryptovirus*, and possesses a double-stranded circular DNA (~ 172 kb) genome. EBV is known as the causative agent of infectious mononucleosis and some malignant diseases such as Burkitt lymphoma and post-transplant lymphoproliferative disorder. However, more than 90% of adults are positive for EBV, leading to the concept that EBV chronically infects humans without causing serious disease [19]. Although EBV preferentially infects human leukocytes, particularly B cells [19], EBV was broadly detected in various tissues (Fig. 2, left). This result may be attributed to the residual blood in each tissue even after the perfusion process, or to tissue-resident cells from the B cell lineage [28].

**Fig. 4:**
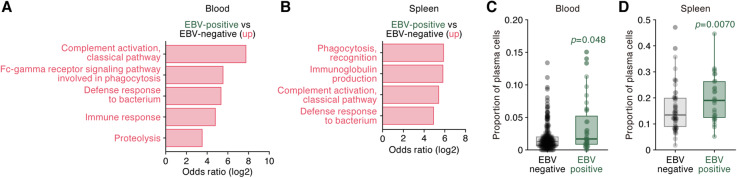
EBV in blood and spleen. **a**, **b** GO enrichment analysis of the upregulated genes in EBV-positive samples in blood (**a**) and spleen (**b**). The terms with statistical significance (FDR < 0.05) are shown. **c**, **d** Increase in the proportion of plasma cells in EBV-positive samples. The results of deconvolution analysis in the blood (**c**) and spleen (**d**) are shown. Each dot indicates the result from respective samples, and the statistical summary of distribution is shown as a box plot. Statistical significance was evaluated by Welch’s *t* test. See also Additional file 8: Figure S3 and Additional file 9: Figure S4

To address the biological impact of EBV infection, we particularly focused on the spleen and peripheral blood, where leukocytes, including B cells, naturally reside or circulate in high abundance. The spleen was the tissue with the highest proportion of EBV-positive samples (Fig. 2, middle). We detected DEGs that were upregulated in EBV-positive samples compared to EBV-negative samples and performed GO enrichment analysis. In both the blood (Fig. 4a) and spleen (Fig. 4b), genes encoding a variety of immunoglobulins were upregulated, leading to enrichment of GO terms such as “complement activation.” This suggested that the abundance of B cells may be increased in EBV-positive samples. To further address this point, we performed deconvolution analysis [39], which estimates the proportion of different immune cell types from bulk RNA-Seq data. The proportion of plasma cells, which produce antibodies in high abundance [40, 41], was significantly increased in EBV-positive samples compared to EBV-negative samples in both the blood (Fig. 4c; see also Additional file 8: Figure S3) and spleen (Fig. 4d; see also Additional file 9: Figure S4). It has been reported that productive EBV replication (also known as “lytic infection”) is initiated during B cell differentiation into plasma cells [42–44]. Moreover, a recent study suggested that EBV infection reprograms the gene expression pattern of infected B cells to resemble plasmablasts and early plasma cells [45]. Our findings correspond well to these experimental observations [42–45] further suggesting the biological validity of our investigations. Moreover, our results raise the possibility that the “healthy” human virome, in particular EBV, may influence the spectrum of B cell lymphoproliferative disorders such as monoclonal gammopathy of unknown significance [46, 47] in different individuals.

### Unrecognized limbic encephalitis associated with HSV-1

HSV-1 is classified into the family *Herpesviridae*, genus *Simplexvirus*, and possesses a double-stranded circular DNA (~ 150 kb) genome. HSV-1 infects a broad range of cell types and tissues and causes cold sores and genital herpes infections [48]. After primary infection, HSV-1 latently infects nerve cells. It sporadically reactivates, leading to recurrent symptoms [48].

**Fig. 5:**
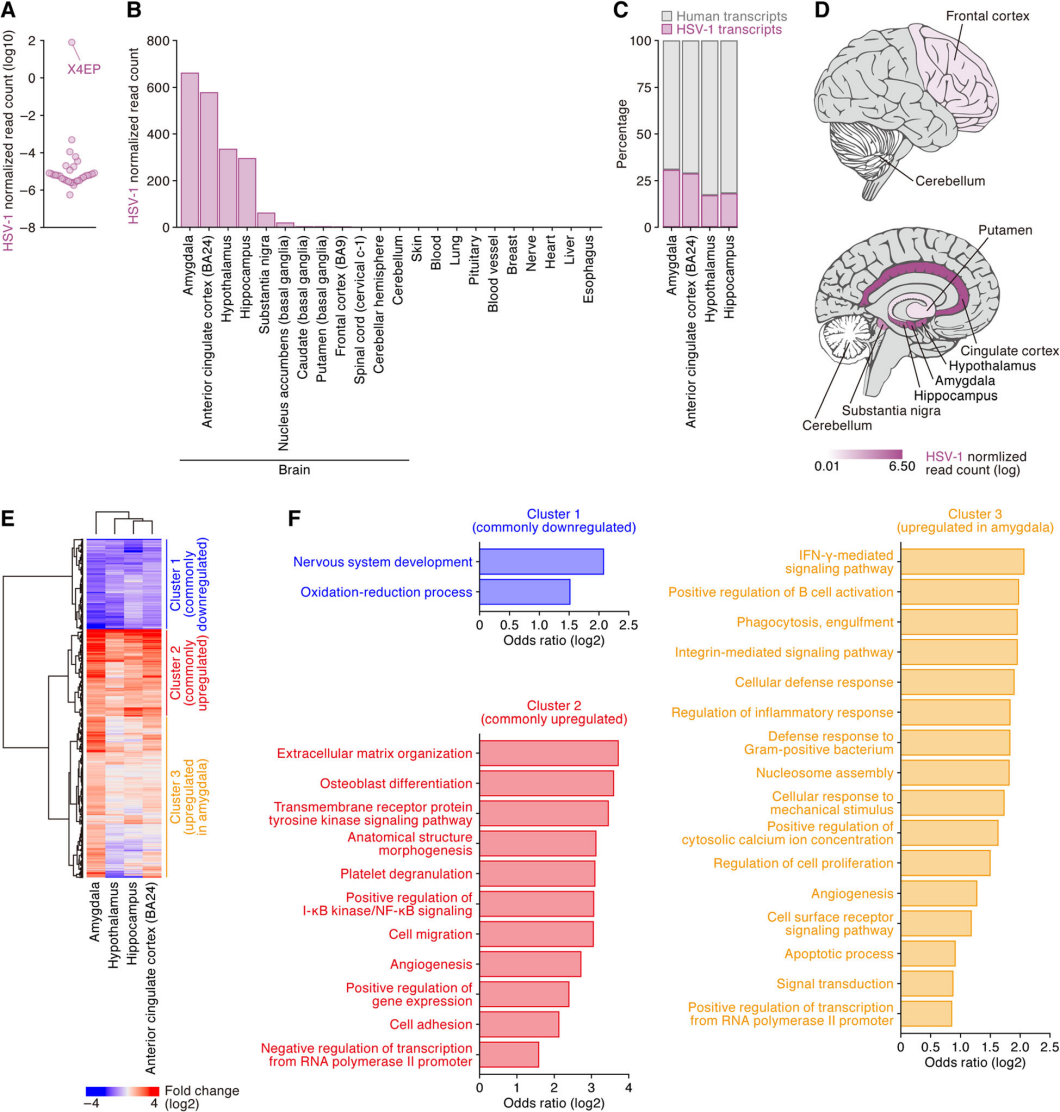
HSV-1 in the brain of an individual. **a** Detection of an individual (sample ID: X4EP) with severe HSV-1 infection. Each dot indicates the normalized read count of HSV-1 in each individual (*n* = 32). Note that HSV-1 reads were undetectable in 515 individuals. **b** HSV-1 transcript abundance across tissues in the individual (sample ID: X4EP). The normalized read counts of HSV-1 are shown. Only the brain was subdivided into each region. BA, Brodmann’s area. **c** Proportion of HSV-1 reads in the total assigned reads. The percentages in the respective brain regions of the individual (sample ID: X4EP) are shown. **d** Anatomical distribution of HSV-1 transcripts in the brain. The normalized expression levels of HSV-1 in the respective brain regions of the individual (sample ID: X4EP) are shown as a brain-shaped heatmap. The regions without RNA-Seq data are shown in gray. **e** Differential gene expression in the brain. DESeq2 was used for feature selection of genes toward downstream analyses. The heatmap contains 1242 DEGs (X4EP sample versus other samples) that were detected in any of the four brain regions (amygdala, hypothalamus, hippocampus, and anterior cingulate cortex). The DEGs were classified into three clusters: cluster 1 (blue), cluster 2 (red), and cluster 3 (orange), mainly consisting of commonly downregulated genes, commonly upregulated genes, and the genes upregulated specifically in the amygdala, respectively. **f** GO enrichment analysis of the three gene clusters. The terms with statistical significance (FDR < 0.05) are shown

HSV-1 encephalitis is a severe and often fatal condition caused by HSV-1 infection [49]. Surprisingly, we detected high levels of HSV-1 transcripts in the brain (Fig. 2, left) of one individual (sample ID: X4EP) (Fig. 5a). As shown in Fig. 5b, HSV-1 transcripts were highly detected in certain brain regions, such as the amygdala, anterior cingulate cortex (also known as Brodmann’s area [BA] 24), hypothalamus and hippocampus, but were faintly detected in other regions, such as the basal ganglia and spinal cord. Reads assigned to HSV-1 accounted more than 20% of the total RNA-Seq reads in these four brain regions (Fig. 5c), which comprises the limbic system (Fig. 5d). Site-specific HSV-1 expression in the brain is consistent with anatomically restricted HSV-1 replication in the brain of patients who exhibit HSV-1 encephalitis [50, 51]. Notably, this subject died a slow death, and ischemic changes are noted on the brain biopsy. It is thus unclear whether the HSV-1 infection present in this subject reflects peri- or even port-mortem reactivation [52], or clinically unrecognized HSV-1 encephalitis present at the time of or even contributing to this subject’s death.

To assess the impact of HSV-1 gene expression in these four brain regions, we compared the expression levels of human genes to those from these same regions in subjects with no HSV-1 expression. DEGs were classified into three clusters: commonly downregulated genes (cluster 1), commonly upregulated genes (cluster 2), and the genes upregulated in the amygdala (cluster 3) (Fig. 5e). GO analysis revealed that neuronal genes (GO term “nervous system development”) were commonly downregulated, while the genes associated with cell activation, inflammation (e.g., GO terms “positive regulation of I-κB kinase/NF-κB signaling” in cluster 2 and “regulation of inflammatory response” in cluster 3), and immune responses (e.g., “platelet degranulation” in cluster 2 and “positive regulation of B cell activation” in cluster 3) were commonly upregulated (Fig. 5f). In particular, the GO term “IFN-γ-mediated signaling pathway” was the highest ranked in cluster 3, and this ontology included ISGs (Fig. 5f). This suggests that HSV-1 expression was present in this subject long enough prior to death to allow for subsequent immune responses. The GTEx annotation for this subject indicates that the cause of death was a hepatic disorder. However, our data may suggest limbic encephalitis due to HSV-1 infection as an alternative cause of death in this subject.

### TTV infection in many human tissues without inducing an IFN response

**Fig. 6:**
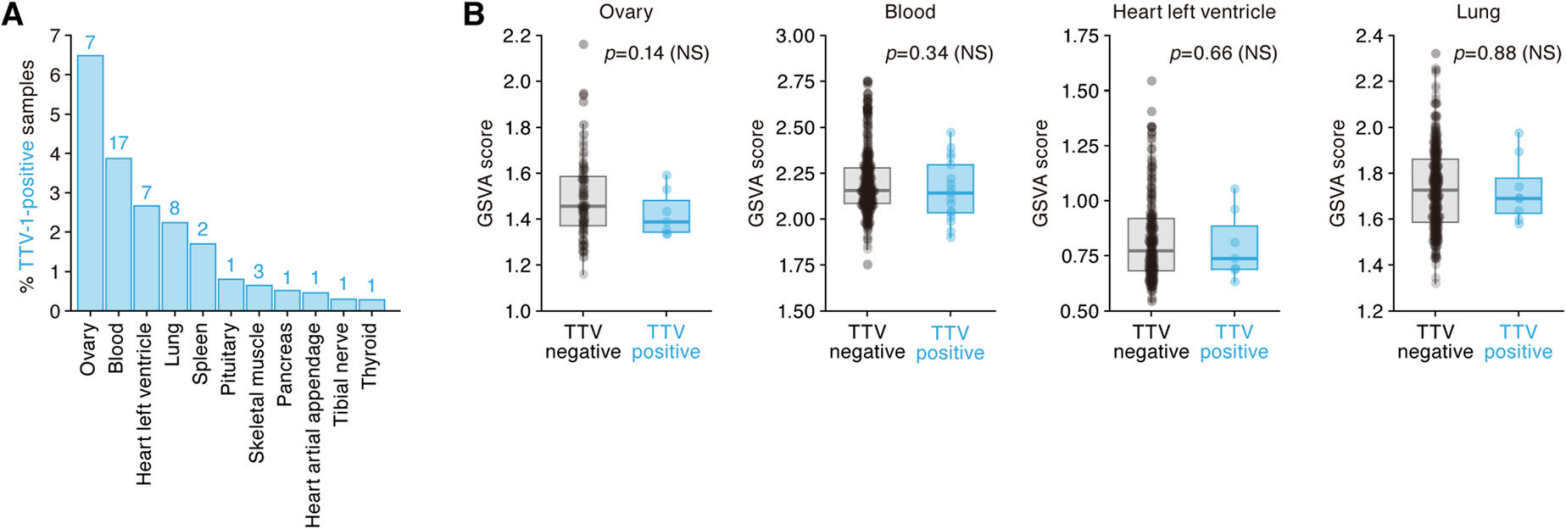
TTV in a variety of tissues in multiple individuals. **a** Percentages of the samples positive for TTV in each tissue. The numbers on the bar graphs indicate the number of samples positive for TTV. **b** The gene set-level expression score (GSVA score) of ISGs in the four tissues of TTV-positive and TTV-negative samples. Each dot indicates the score of each sample. Statistical significance is evaluated by Welch’s *t* test. NS, no statistical significance

Torque teno virus (TTV) was recently classified into the family *Circoviridae*, genus *Anellovirus*, by the International Committee on Taxonomy of Viruses (ICTV) in 2011 [53]. TTV is the first recognized human virus with a single-stranded circular DNA (~ 3.8 kb) genome [54]. Although this virus was first found in a patient with non-A to G hepatitis in Japan [55], its association with diseases is poorly understood [54]. As shown in Fig. 6a, TTV was detected in a variety of tissues in multiple individuals. TTV is a single-stranded DNA virus, and human Toll-like receptor 9 is a PRR that senses single-stranded DNA and induces IFN production [56, 57]. Therefore, we hypothesized that TTV infection is associated with increased ISG expression, as observed for HCV (Fig. 3) and HSV-1 (Fig. 5). To address this hypothesis, we chose four tissues (ovary, blood, heart [left ventricle], and lung) with a high percentage of TTV-positive samples (Fig. 6a). However, upregulation of ISGs in association with TTV infection was not observed (Fig. 6b). Our findings expand on those from a DNA-based virome survey [30] to suggest that TTV not only infects, but is transcribed in a variety of tissues in a large fraction of people. Moreover, we show that it does so without triggering IFN production. In light of the known association with increased TTV viremia after transplantation and immunosuppression [58], these results raise the possibility that an immune pathway other than IFN may be responsible for the control of this virus. Although it remains unclear whether TTV infection potentially associates with certain diseases, our findings suggest that TTV is a “commensal” virus that is easily detectable at the level of RNA expression in a variety of human tissues with relatively high prevalence.

### Strong association of a human gene expression pattern with the presence of HHV-7 transcripts in the stomach

**Fig. 7:**
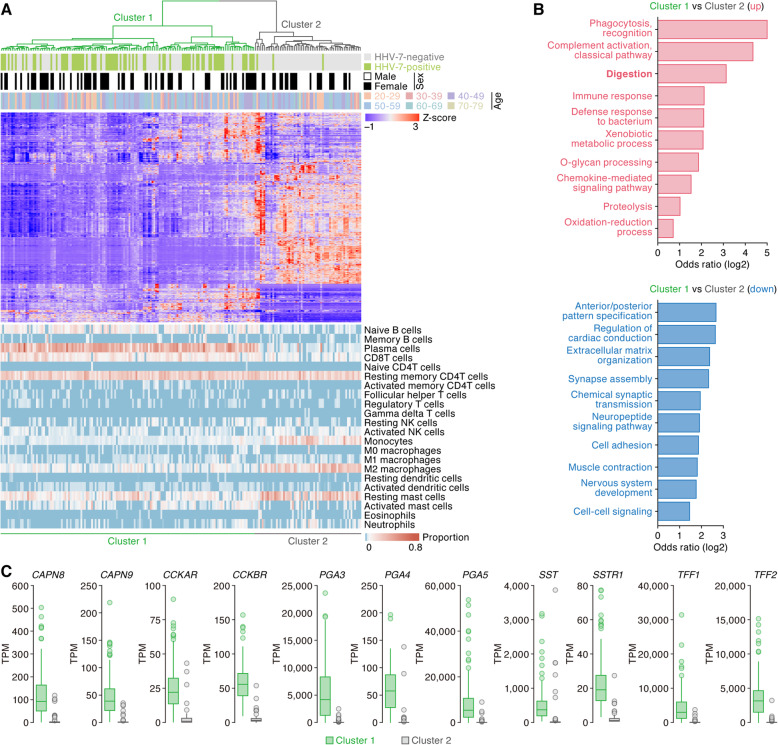
Potential effect of HHV-7 infection on human gene expression in the stomach. **a** Association of the human gene expression pattern with HHV-7 infection in the stomach. Stomach samples (*n* = 203) were classified into the two clusters based on the 1000 most highly expressed human genes. (Top) The relative expression levels of human genes are shown as a heatmap. (Middle) Distribution of the samples positive for HHV-7 (*n* = 78). Note that HHV-7-positive samples accumulated in cluster 1. (Bottom) Deconvolution analysis of human leukocytes residing in the stomach. The proportions of the respective human leukocytes are shown as a heatmap. **b** GO enrichment analysis of the DEGs between clusters 1 and 2. The top 10 terms significantly upregulated (top) and downregulated (bottom) in cluster 1 compared to those in cluster 2 are shown. **c** Differential expression of the genes associated with the digestion process. The expression levels of 11 genes associated with the digestion process (*CAPN8*, *CAPN9*, *CCKAR*, *CCKBR*, *PGA3-5, SST, SSTR1*, *TFF1*, and *TFF2*) in clusters 1 and 2 are shown as TPM

HHV-7 is classified into the family *Herpesviridae*, genus *Roseolovirus*, and possesses a double-stranded circular DNA (~ 153 kb) genome [32]. This virus is known as one of the causative agents of roseola infantum (also known as exanthema subitum), a usually mild illness of infants [32]. Surprisingly, we detected HHV-7 transcripts specifically in the stomach (Fig. 2, left), and 37.4% (76 out of the 203 samples) of the stomach samples were positive for HHV-7 (Fig. 2, middle and right). Our findings expand on those from a recent DNA-based virome survey [59] and suggest that HHV-7, a member of roseolovirus, not only infects, but is transcribed in the stomach. Using unsupervised clustering of human gene expression patterns, stomach samples were classified into two clusters according to the human gene expression patterns (Fig. 7a, top). HHV-7-positive samples were strikingly enriched in cluster 1 (Fig. 7a, middle; 73 out of the 76 HHV-7-positive samples; 96.1%). These results suggest that the HHV-7 transcription status is strongly associated with the global human gene expression pattern in the stomach. The difference of global human transcriptome between clusters 1 and 2 may be due to the different anatomical regions, and HHV-7 may predominantly infect the stomach region categorized as cluster 1.

HHV-7 preferentially infects CD4^+^ T cells [32]. The results of deconvolution analysis [39] of the transcriptome of both HHV-7-positive and HHV-7-negative samples were consistent with the presence of resting memory CD4^+^ T cells in the stomach (Fig. 7a, bottom). Additionally, transcripts attributable to plasma cells were relatively abundant in cluster 1, while those attributable to myeloid cells (monocytes and M2 macrophages) and mast cells were abundant in cluster 2 (Fig. 7a, bottom). These findings suggest that HHV-7 infection is associated with the pattern of leukocytes residing in the stomach. We further performed GO analysis on the DEGs between these two clusters. In addition to the GO terms associated with tissue-resident leukocytes (e.g., “phagocytosis” and “immune response”), GO terms such as “digestion” were highly ranked in the upregulated genes in cluster 1 compared to those in cluster 2 (Fig. 7b). In fact, the expression levels of some genes encoding enzymes and proteins that play critical roles in digestion in cluster 1 were significantly higher than those in cluster 2 (Fig. 7c). For instance, calpains (*CAPN8* and *CAPN9*) [60] and pepsinogens (*PGA3–5*) [61] digest proteins and peptides, and the signals mediated by cholecystokinin receptors (*CCKAR* and *CCKBR*) help the digestion of proteins and lipids [62]. Somatostatin (*SST*) and its receptor (*SSTR1*) control the secretion of gastric acids [63], and trefoil factors (*TFF1* and *TFF2*) help protect and repair the gastrointestinal mucosa [64]. These genes are expressed in stomach cells (i.e., secretory epithelial cells) but not in leukocytes. Thus, our results suggest that HHV-7 infection may be associated with increased expression of the transcripts important in the function of the stomach (Fig. 7b). Another possibility is that HHV-7 is highly transcribed in the stomach region where digestive genes are highly expressed. More study of the potentially mutualistic relationship between HHV-7 and humans influencing digestive function will be needed.

### Effect of local virus infections on systemic antiviral immunological homeostasis

**Fig. 8:**
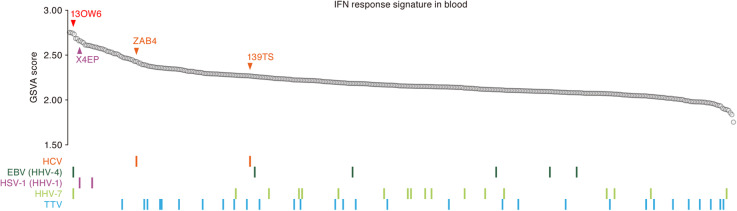
Effect of virus infections on IFN responses in body-circulating blood. (Top) The gene set-level expression score (GSVA score) of ISGs in the blood. The colored arrows with sample IDs indicate the positions of the samples positive for HCV (orange) and HSV-1 (purple). The sample positive for both EBV and HHV-7 (sample ID: 13OW6) is indicated by a red arrow. (Bottom) Individuals positive for respective viruses. “Positive” means that the virus abundance is more than the upper quartile

In Fig. 2, we summarized the tissue-level tropism of 39 viruses present in the human virome. We found upregulation of ISGs, a hallmark of the human responses against virus infections, in certain tissues with evidence of viral transcription, including HCV in the liver (Fig. 3) and HSV-1 in the brain (Fig. 5). However, it was unclear whether abundant viral transcription and likely productive viral infection we observed localized to certain tissues (e.g., liver and brain) affected systemic immunological homeostasis in the human body. To investigate this issue, we evaluated the magnitude of IFN responses in the blood, circulating throughout the human body, as the gene set-based expression score of ISGs [65]. As shown in Fig. 8, the magnitude of ISG expression in the blood varied in the respective individuals. Interestingly, the individuals that exhibited HCV infection in the liver (Fig. 3) and HSV-1 in the brain (Fig. 5) exhibited relatively increased levels of ISG expression even in the blood, where the transcription of these viruses was hardly detected. These results suggest that local tissue-specific expression of these viruses may have triggered an IFN response in the blood, perturbing systemic immunological homeostasis. On the other hand, the sole infection of EBV, HHV-7, and TTV was not associated with the magnitude of ISG expression in the blood (Fig. 8). These findings suggest that the potential to trigger innate immune responses (e.g., IFN production) may be dependent on the type of virus or the chronicity of viral infection. However, a sample positive for both EBV and HHV-7 (sample ID: 13OW6) exhibited a very high GSVA score of ISGs in the blood (Fig. 8), which suggests the possibility that co-infection of these viruses may synergistically trigger an IFN response in the blood.

## Discussion

In this study, we comprehensively addressed the human virome in a variety of human tissues through meta-transcriptomic analysis. We found that the human virome includes several viruses “hidden” by expression/replication in tissues inside the human body without being abundant in the peripheral blood. As expected, different individuals and different tissues harbor distinct virome. We described some expected (e.g., the upregulation of ISGs by HCV infection) and unexpected (e.g., the association of HHV-7 infection with higher expression of digestive genes in the stomach) associations with the presence of viral RNAs. Collectively, these findings strongly suggest that our analytical pipeline is useful to reveal the potential impacts of viral infections on human health. Moreover, in our recent study [66], we used the same analytical pipeline and detected the expression of inherited chromosomally integrated HHV-6 from the GTEx subjects, which were positive for this integrated virus reported in another study [67]. This further strengthens the confidence of our approach. However, some improvements to the analytical pipeline could be beneficial for subsequent work to define the virome. First, since we employed a quantification-oriented analytical pipeline, we probably failed to detect viruses that are unknown or highly divergent from the reference genome sequences. To find such viruses in silico, de novo assembly techniques followed by sequence similarity searches on translated nucleotide databases (i.e., tBLASTn) should be employed. Second, here we excluded retroviruses, including HIV, from investigation because abundant sequences derived from endogenous retroviruses in the human genome were detected, and it was difficult to distinguish them from authentic exogenous retroviruses. If retroviruses, including HIV, are targeted, the analytical pipeline should be modified. Third, as implied in a previous report [27], relatively low abundant sequences might be attributed to the contamination from commercial reagents and the environment or mistakes in the demultiplexing of NGS reads. To exclude the effect of cross-contamination, our analytical pipeline included several filters (see Fig. 1 and the “Methods” section). Nevertheless, we detected a low number of reads mapped to Lassa virus in some samples (Fig. 2). Lassa virus is the causative agent of the often fatal Lassa hemorrhagic fever and is prevalent only in Africa (reviewed in [68]); the GTEx samples were collected in the USA. We are unable to completely exclude the possibility that the GTEx samples positive for Lassa virus transcripts came from subjects asymptomatically infected with this virus. However, these reads mapped only one (L segment) out of the viral two segments (see the “Methods” section and Additional file 10: Figure S5). Moreover, although Lassa virus infection strongly induces IFN responses [69], ISGs were not upregulated in Lassa virus-positive specimens (data not shown). Since we did not find supportive biological results suggesting that the reads of Lassa virus we detected are authentic, we did not deeply focus on this virus in this study. To deeply survey the human virome with high accuracy, experimental verification of the presence of viruses suggested by reads at very low is required.

## Conclusion

Here we performed integrative analyses on the association of virus infections with the human transcriptome at tissue-level resolution. Our results suggest that the human virome potentially influences human health, not only disease. While our results document the healthy human virome in new detail, they will surely motivate even more granular investigations of this virome in the future, both as described above and at the resolution of single cells. This could allow screening for new viral pathogens and simultaneous definition of viral tropism. Future investigations using NGS data obtained from both disease-affected and healthy subjects will thus enable detection of currently hidden associations between the human virome and human health and disease.

## Methods

### Dataset

Pair-ended, poly A-enriched RNA-Seq data provided by GTEx (version 7.p2) were analyzed. Information of the analyzed RNA-Seq dataset is summarized in Additional file 1: Table S1. The raw fastq files were downloaded from the Sequence Read Archive (SRA) using SRA Toolkit (version 2.8.2-1). Only data with the expected sequencing length (i.e., “AvgSpotLen” column in Additional file 1: Table S1) less than 200 bp were analyzed (i.e., long-read sequencing data were excluded).

Reference genome sequences of viruses registered in the NCBI Viral Genomes Resource [70] were downloaded via Batch Entrez, accessed 13 January 2019. Only viruses whose hosts are registered as invertebrates, vertebrates, and humans were analyzed. Information on the viral genome sequences analyzed in the present study is summarized in Additional file 11: Table S6.

Sequences of prokaryotic representative genomes were downloaded from the NCBI RefSeq Database [71], accessed 14 February 2019. Information of the prokaryotic genome sequences used in the present study is summarized in Additional file 12: Table S7.

Image data were downloaded manually from Histology Viewer on GTEx Portal [29]. Then, representative digital images of each data were collected using Aperio Imagescope software (version 12.3.3.5048, Leica Biosystems).

As sources of functional gene sets, “GO biological process,” “GO cellular component,” “MSigDB canonical pathway,” and “InterPro” were used. “GO biological process” and “GO cellular component” were obtained from the GO Consortium [72, 73] (GO validation date: 30 August 2017), “canonical pathway” was from MSigDB [74, 75] (version 6.1), and “InterPro” was from BioMart on the Ensembl website [76] on 13 February 2018.

### Construction of custom sequence databases

A viral genomic sequence database was constructed as follows. First, of the viral sequences, sequence regions that highly resemble the human or bacterial genomes were masked (i.e., replaced by the sequence “NNN …”). The sequence regions to be masked were determined by a local sequence similarity search using BLASTn (in BLAST+ version 3.9.0) [77]. The word size and *E* value parameters were set at 11 and 1.0e−3, respectively. As sources of the human and bacterial genome sequences, the human reference genome (GRCh38/hg38) and the prokaryotic representative genomes were used, respectively. Second, redundant viral sequences (i.e., sequences disclosing > 90% global sequence identity with each other) were concatenated. The sequence identity was calculated by Stretcher software (in EMBOSS version 6.6.0.0) [78]. The viral sequences included in the viral sequence database are summarized in Additional file 11: Table S6. In addition, a sequence database comprising (1) the human reference genome, (2) the prokaryotic representative genomes, and (3) the custom viral genomes prepared above was also constructed.

### Quantification of human gene expression

Low-quality sequences in RNA-Seq fragments (i.e., a set of paired RNA-Seq reads) were trimmed using Trimmomatic (version 0.36) [79] with the option “SLIDINGWINDOW:4:20.” RNA-Seq fragments were mapped to the human reference genome (GRCh38/hg38) using STAR (version 2.6.0c) [80] with the GENCODE gene annotation (version 22) [81]. STAR was run using the same options and parameters as those used in the GDC mRNA Analysis Pipeline. RNA-Seq fragments mapped on the exons of genes were counted using Subread featureCounts (version 1.6.3) [82] with GENCODE gene annotation. The option “fracOverlap” was set at 0.25. RNA-Seq fragments assigned to multiple features were not counted. Among the GENCODE gene annotations, only the genes assigned to any biological features (i.e., the genes that are registered in any of the functional gene sets [described in the above section]) were used for analysis. The expression level of human genes was normalized as transcripts per million (TPM) [83].

### Extraction of RNA-Seq fragments disclosing similarity to viral sequences

The analytical pipeline is illustrated in Fig. 1. First, RNA-Seq fragments that were not properly mapped to the human reference genome were extracted using the “samtools view” [84] command with the options “-f 12 -F 256.” Second, a 1st BLASTn search of the unmapped RNA-Seq reads was performed on the viral genome sequence database prepared above. The word size and *E* value parameters were set at 11 and 1.0e−10, respectively. Regarding the BLAST-hit reads, a 2nd BLASTn search was performed on the database comprising the human, bacterial, and viral genome sequences prepared above. RNA-Seq fragments were assigned to the top-hit feature according to the bit score. In the case of a tie, the reads were discarded. If a set of paired reads was assigned to the distinct features, the reads were discarded. RNA-Seq fragments assigned to respective viruses were counted in each RNA-Seq dataset, and a count matrix of the viral RNA-Seq fragments was generated.

### Filtering low-reliability viral hits

From the count matrix of the viral RNA-Seq fragments, viruses in which the total count was less than 5 were excluded. Viruses that were detected only in a single experimental batch (SMGEBTCH; Additional file 1: Table S1) were also excluded. In addition, we excluded viruses for which only a limited portion of the viral genome was covered by the RNA-Seq fragment hits. We divided equally a viral genome sequence into 10 regions, and we excluded the virus if RNA-Seq fragments hit only < 5 out of the 10 regions in the viral genome. For example, Lassa virus satisfied this filtering criteria, but Pepper chlorotic spot virus did not because of only limited fraction assigned with reads (Additional file 10: Figure S5). Finally, we manually curated the list of detected viruses. *Retroviridae* viruses were removed because there is a possibility of cross-contamination from endogenous retroviral sequences. “*Autographa californica* nucleopolyhedro virus (accession no. NC_001623.1)” was removed because there is a possibility of cross-contamination from baculovirus vectors. “Chimpanzee alpha-1 herpesvirus strain 105640” (accession no. NC_023676.1) was also removed because of the suspicion of misshit of HSV-1 transcripts: “Chimpanzee alpha-1 herpesvirus strain 105640” was detected only in one individual who held an extremely large amount of HSV-1 reads (sample ID: X4EP, in Fig. 5a). The final set of detected viruses, consisting of 39 viral species, is listed in Additional file 2: Table S2. For illustration and analysis, various subtypes of detected TTVs, human papilloma viruses (HPVs), and Hubei picornavirus were unified as shown in Additional file 2: Table S2.

GTEx provides RNA-Seq data of lymphocytes immortalized by EBV infection (i.e., lymphoblastoid cells [LCLs]). Since it is suspected that there is read contamination from LCL samples in the sequencing step, we did not measure the EBV transcript abundance in the samples that had the same experimental batch (SMGEBTCH) as the LCL samples. As a result, the EBV transcript abundance was measured only in 3031 out of the 8991 samples. Additionally, as shown in Fig. 5a, extremely high levels of HSV-1 transcripts were detected in the samples of the subject “X4EP.” Therefore, we did not measure the HSV-1 transcript abundance in the samples that had the same experimental batch as the X4EP samples. As a result, the HSV-1 transcript abundance was measured only in 8879 of the 8991 samples.

### Quantification of viral transcript abundances

To normalize viral transcript abundance, the number of RNA-Seq fragments assigned to a certain virus was divided by the total number of RNA-Seq fragments assigned to any human genes, the length of the human genome sequence, and the length of the viral genome sequence, as described in a previous study [27]. The RefSeq annotation recodes many subtypes of TTV, HPV, and Hubei picornavirus. In each virus species, viral RNA abundances of the subtypes were summed, and the total viral abundance was used for analyses.

### Comparison of the performance of analytical pipelines

To evaluate the performance of our pipeline, we used three published pipelines: Kraken (version 2.0.8) [35], CLARK (version 1.2.6.1) [36], and Kaiju (version 1.7.3) [37]. As the viral sequence database, NCBI Viral Genomes Resource [70] was downloaded through the command implemented in each pipeline (downloaded 24 February 2020). From 136 liver samples, the number of reads assigned to HCV taxonomy (taxid 11103 or 41856) was quantified by three pipelines with default parameters and compared to our pipeline result (Additional file 7: Figure S2).

### Repetitive sequence annotation in EBV, HHV-6, and HHV-7

To annotate telomeric/repetitive regions on herpesvirus genomes, we used a web-based RepeatMasker software (version open-4.0.9) [85]. From the search results, genomic regions annotated as “Simple_repeat” class were regarded as repetitive regions. Of these, regions annotated as “(CTAACC)n” were regarded as telomeric regions (Additional file 4: Figure S1).

### Differential gene expression analysis and functional annotation

Differential expression analysis was performed by DESeq2 (version 1.24.0) [86]. Differentially expressed genes were detected according to the following criteria: false discovery rate (FDR) < 0.05 and the absolute value of log2-transformed fold change > 1. Gene ontology enrichment analysis (Figs. 3e, 4a, b, 5f, and 7b) was performed by an overlapping-based method with Fisher’s exact test. FDR correction was performed using the Benjamini-Hochberg method. Redundant gene sets were summarized using REViGO [87]. Only the results for the GO biological process are shown. Immune cell composition in a “bulk” tissue sample (Figs. 4c, d, 7a, Additional file 8: Figure S3, and Additional file 9: Figure S4) was estimated from the gene expression data using CIBERSORT (version 1.06) [39]. The TPM-normalized gene expression matrix and LIM-22 dataset in CIBERSORT were used as mixture and signature matrices, respectively. As the gene set-level expression score of ISGs, that of the “type I IFN signaling pathway (GO: 0060337)” calculated by Gene Set Variation Analysis (GSVA) software [65] was used (Figs. 6b and 8).

### Visualization

All heatmaps were drawn by the R package ComplexHeatmap (version 1.17.1) [88]. HSV-1 expression annotation to brain regions (Fig. 5d) was performed by the R package cerebroViz (version 1.0) [89].

## Supplementary information

**Additional file 1: Table S1** Information on the RNA-Seq dataset used in this study.

**Additional file 2: Table S2** List of the 39 viral species detected in this study.

**Additional file 3: Table S3** Read count of the respective 39 viral species detected in this study.

**Additional file 4: Figure S1** Read coverage on EBV, HHV-6 and HHV-7 genomes.

**Additional file 5: Table S4** List of the genomic positions of the masked regions in each viral genome.

**Additional file 6: Table S5** Information on the GTEx samples.

**Additional file 7: Figure S2** Comparison of our analytical pipeline with the other pipelines.

**Additional file 8: Figure S3** Deconvolution analysis in the blood of EBV-positive and -negative subjects.

**Additional file 9: Figure S4** Deconvolution analysis in the spleen of EBV-positive and -negative subjects.

**Additional file 10: Figure S5** Read mapping to Lassa virus segment L and Pepper chlorotic spot virus segment L.

**Additional file 11: Table S6** Information on the viral genome sequences used in this study.

**Additional file 12: Table S7**. Information on the prokaryotic genome sequences used in this study.

## Data Availability

The data, associated protocols, code, and materials in this study are available at reference [91].

## Abbreviations

AAV: Adeno-associated virus
AIDS: Acquired immunodeficiency syndrome
BA: Brodmann’s area
CMV: Cytomegalovirus
dbGaP: Database of Genotypes and Phenotypes
DEG: Differentially expressed gene
EBV: Epstein-Barr virus
FDR: False discovery rate
GBV-C: GB virus C
GO: Gene ontology
GSVA: Gene Set Variation Analysis
GTEx: Genotype-Tissue Expression
HCoV: Human coronavirus
HCV: Hepatitis C virus
HCV: Hepatitis G virus
HHV-1: Human herpesvirus 1
HHV-3: Human herpesvirus 3
HHV-4: Human herpesvirus 4
HHV-5: Human herpesvirus 5
HHV-6A: Human herpesvirus 6A
HHV-6B: Human herpesvirus 6B
HHV-7: Human herpesvirus 7
HIV: Human immunodeficiency virus
HPV: Human papilloma virus
HSV-1: Herpes simplex virus type 1
ICTV: International Committee on Taxonomy of Viruses
IFN: Interferon
ISG: Interferon-stimulated gene
LCL: Lymphoblastoid cell
NGS: Next-generation sequencing
NS: No statistical significance
PRR: Pathogen recognition receptor
RNA-Seq: RNA-sequencing
RSV: Respiratory syncytial virus
TPM: Transcripts per million
TTV: Torque teno virus
VZV: Varicella-zoster virus
