# Supplementary material for: A tissue level atlas of the healthy human virome
Source: BMC Biol. 2020 Jun 4;18:55. doi: 10.1186/s12915-020-00785-5 (PMC7269688; doi:10.1186/s12915-020-00785-5)
Supplement: Supplementary file 4 — Additional file 4: Figure S1 Read coverage on EBV, HHV-6 and HHV-7 genomes. [file 12915_2020_785_MOESM4_ESM.pdf]

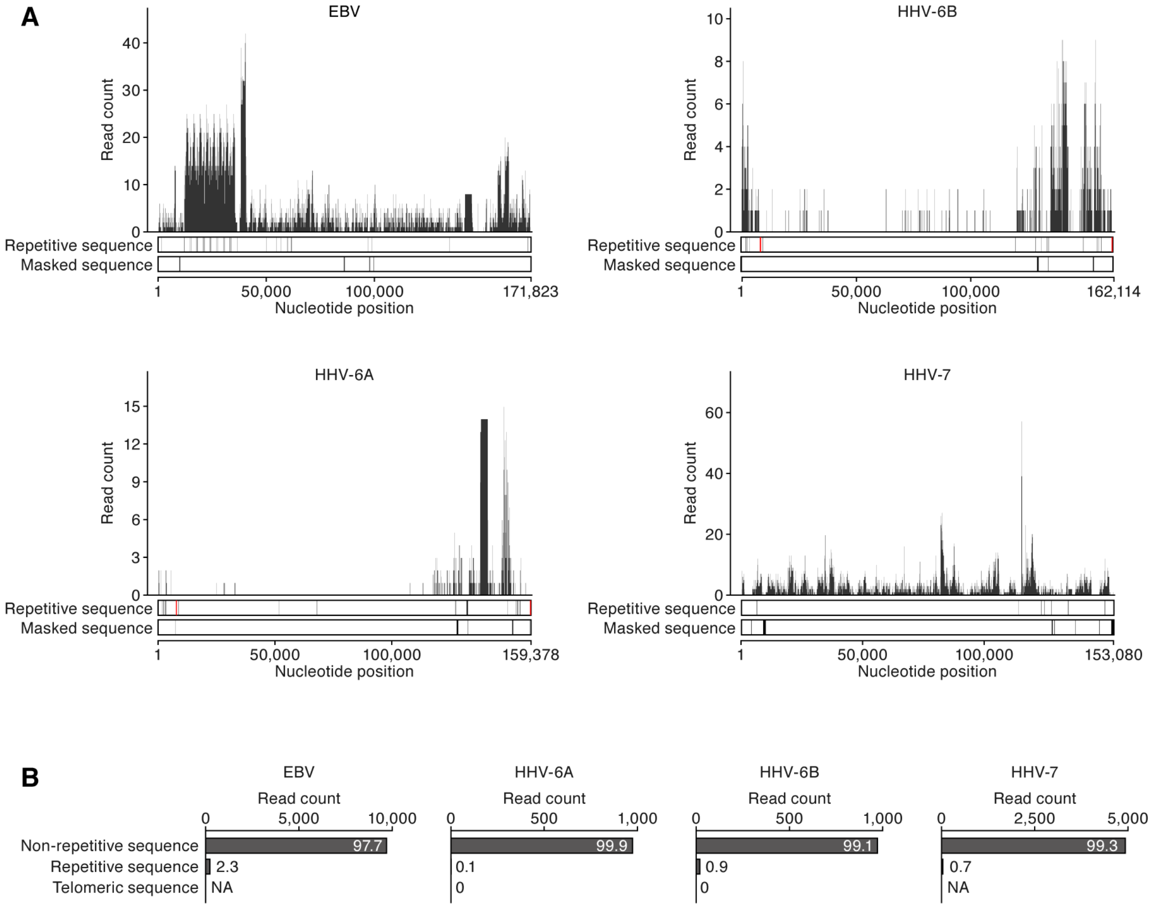

Additional file 4: Figure S1. Read coverage on EBV, HHV-6, and HHV-7 genomes. (A) Read coverage on herpesvirus genomes. Results of the reads collected from all samples are shown. The regions of repetitive sequence and masked sequence in each viral genome are summarized at the bottom of graph. Telomeric repeats are indicated in red. (B) Proportion of the reads assigned to telomeric/repetitive sequences. The number in the graph indicates the percentage of read counts mapped to each region. NA, not applicable. See also **Additional file 5: Table S4.**
