# Supplementary material for: A tissue level atlas of the healthy human virome
Source: BMC Biol. 2020 Jun 4;18:55. doi: 10.1186/s12915-020-00785-5 (PMC7269688; doi:10.1186/s12915-020-00785-5)
Supplement: Supplementary file 7 — Additional file 7: Figure S2 Comparison of our analytical pipeline with the other pipelines. [file 12915_2020_785_MOESM7_ESM.pdf]

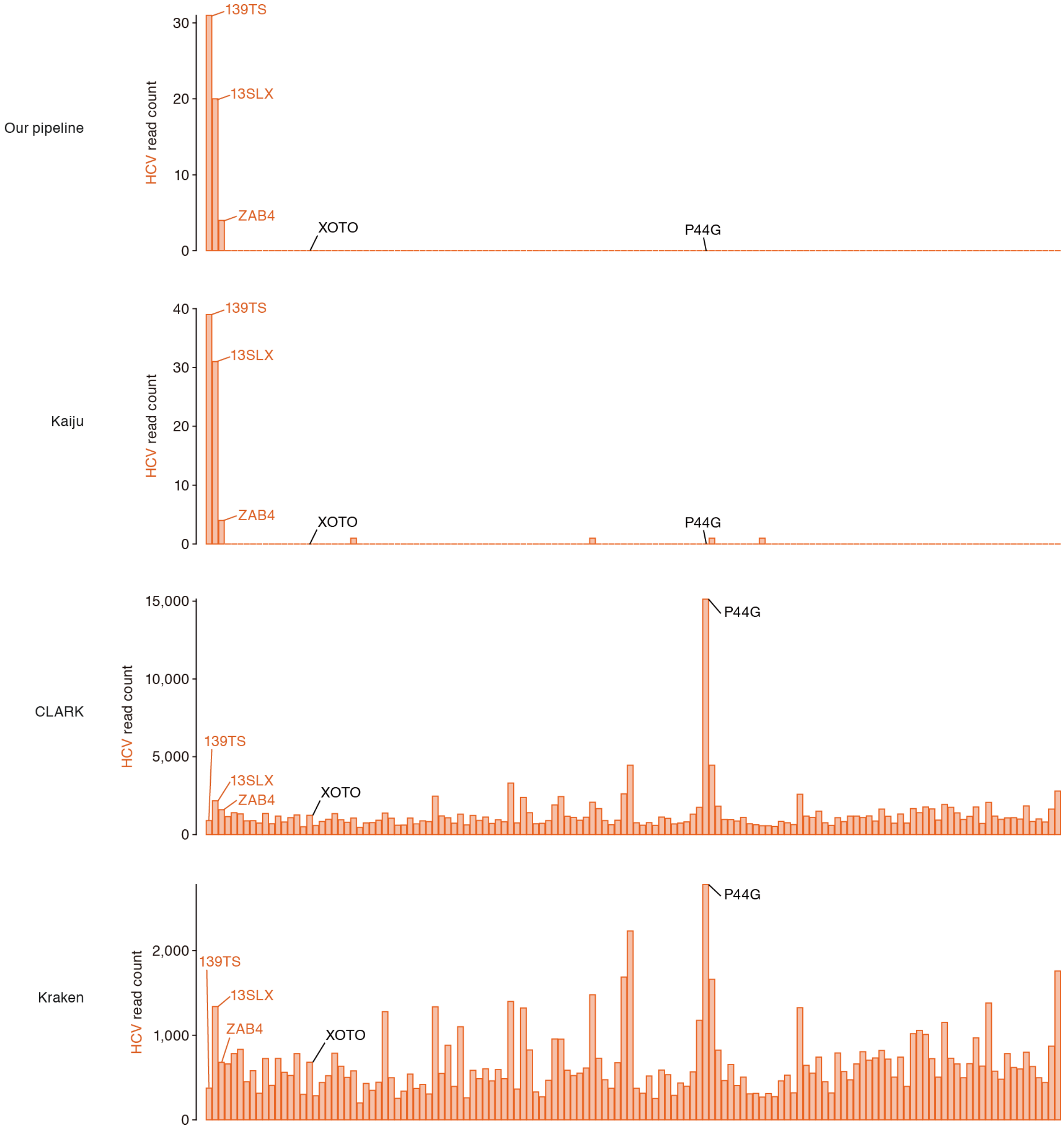

Additional file 7: Figure S2. Comparison of our analytical pipeline with the other pipelines.

The amounts of HCV reads in each liver sample (n=136) detected by four pipelines: our pipeline, Kaiju, CLARK, and Kraken, are shown. Each bar column in each panel indicates the same sample. The liver samples shown in Fig. 3B are indicated in the figure.
