# Supplementary material for: A tissue level atlas of the healthy human virome
Source: BMC Biol. 2020 Jun 4;18:55. doi: 10.1186/s12915-020-00785-5 (PMC7269688; doi:10.1186/s12915-020-00785-5)
Supplement: Supplementary file 9 — Additional file 9: Figure S4 Deconvolution analysis in the spleen of EBV-positive and -negative subjects. [file 12915_2020_785_MOESM9_ESM.pdf]

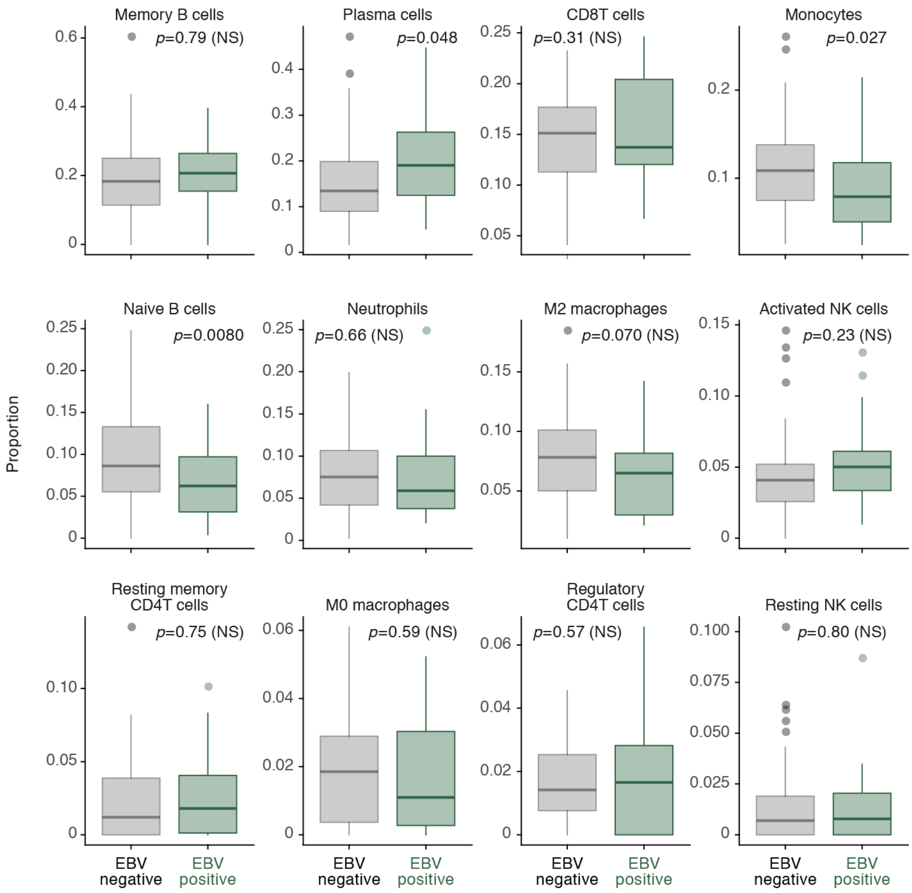

Additional file 9: Figure S4. Deconvolution analysis in the spleen of EBV-positive and -negative subjects.

Distributions of the proportions of top 12 cell types with high proportion in spleen are summarized. Each dot indicates the result from respective samples, and the statistical summary of distribution is shown as a box plot. Statistical significance was determined by Welch’s *t* test. Note that the panel of "plasma cells" is identical to that in **Fig. 4D**.
