# Supplementary material for: A tissue level atlas of the healthy human virome
Source: BMC Biol. 2020 Jun 4;18:55. doi: 10.1186/s12915-020-00785-5 (PMC7269688; doi:10.1186/s12915-020-00785-5)
Supplement: Supplementary file 10 — Additional file 10: Figure S5 Read mapping to Lassa virus segment L and Pepper chlorotic spot virus segment L. [file 12915_2020_785_MOESM10_ESM.pdf]

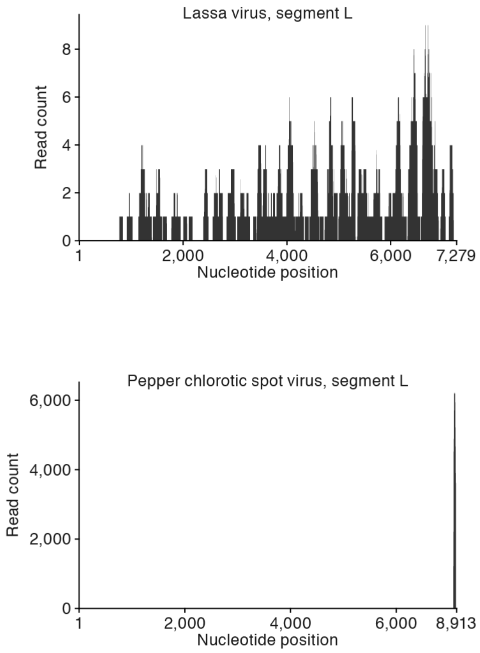

Additional file 10: Figure S5. Read mapping to Lassa virus segment L and Pepper chlorotic spot virus segment L.

Coverages of the reads of Lassa virus and Pepper chlorotic spot virus from all samples are shown. Note that the reads of these viruses are only mapped to a viral segment, segment L.
